# Editorial: Plant microbiome: interactions, mechanisms of action, and applications, volume III

**DOI:** 10.3389/fmicb.2025.1696341

**Published:** 2025-10-07

**Authors:** Alok Kumar Srivastava, Prem Lal Kashyap, Gustavo Santoyo, George Newcombe

**Affiliations:** ^1^Indian Council of Agricultural Research (ICAR)-National Bureau of Agriculturally Important Microorganisms, Mau, India; ^2^Indian Council of Agricultural Research (ICAR)-Indian Institute of Wheat and Barley Research (IIWBR), Karnal, India; ^3^Instituto de Investigaciones Químico-Biológicas, Universidad Michoacana de San Nicolás de Hidalgo, Morelia, Mexico; ^4^Department of Forest, Rangeland and Fire Sciences, University of Idaho, Moscow, ID, United States

**Keywords:** AMF, PGPR, plant–microbe interactions, sustainable agriculture, *Trichoderma*

Terrestrial plants are of unique evolutionary and ecological importance (Margulis and Sagan, 1997). At present, approximately 400,000 plant species comprise nearly 80% of the world's living biomass (Bar-On et al., 2018). Their symbionts, including representatives of fungi, bacteria and other microorganisms, are equally remarkable (Gray, 2017). Microbes inhabit nearly all plant tissues, both inside and out (Barnes et al., 2025); collectively, they are referred to as the plant microbiome. The interactions within and among these microbes, and between microbes and plants, are central to terrestrial life, shaping individual plant phenotypes and driving the functioning of entire ecosystems (Barnes et al., 2025).

This Research Topic focuses on the interactions of plants with fungi and bacteria. Fungi vary in their interactions with plants, from obligate parasites (e.g., rust fungi, order Uredinales) to obligate mutualists without which certain plants cannot survive (e.g., some mycorrhizal interactions). Some fungi are even parasitized by plants (Bidartondo, 2005). The functional diversity of plant-fungal interactions is striking. Fungi, themselves, “probably evolved from a line of fungus-like protists that absorbs food directly from the living or dead bodies of algae, plants, and animals; and fungi seems to have coevolved with plants in the move to the land” (Margulis and Sagan, 1997). These authors argued that without their symbionts, plants might never have emerged and evolved on land: “dry land was as hostile an environment for plants as the moon is for us.”

Today, drought and salinity represent major constraints on crop yields (Verma et al., 2021). The contributions to this research topic primarily address research on microbes that improve the growth and yield of economically important plants affected by drought and salinity (Delaux and Schornack, 2021). Knowledge of endophyte applications to crops is more advanced than in any other sector of agriculture. However, even with crop plants, the most effective endophytes may never be identified, and even when they are their performance in a given crop system may fall short of optimal outcomes in the near future.

Advances in plant–microbe research reveal the critical role of microbiomes in maintaining plant health, productivity, and resilience in both ecological and agricultural environments. A central theme across the articles in *Plant Microbiome: Interactions, Mechanisms of Action, and Applications, Volume III*, is the idea that plants and their microbiomes are integrated holobionts and understanding their interactions is key to solving problems of food security, environmental stress, loss of biodiversity, and the sustainable use of bioresources. Together, the articles cover staple cereals, legumes, fruit trees, medicinal herbs, forest ecosystems, and desert shrubs, painting a tapestry of how microbiomes influence plant function, environmental tolerance, and even the integrity of medicinal compounds.

An overarching theme throughout this collection of works was the untapped microbial richness within medicinal and endangered plants. Research on *Elephantorrhiza elephantina*, a traditionally valued but declining medicinal herb in southern Africa, illustrates how a combination of both next-generation sequencing and culturing strategies can uncover concealed microbial symbionts in plant tissues. Predominant taxa such as *Pseudomonas, Microbacterium oxydans*, and *Stenotrophomonas maltophilia* were not only detected but also found to be bioactive metabolite producers, with high antimicrobial activity against a wide array of bacterial pathogens (Tlou et al.). Their demonstrated production of antimicrobial metabolites revealed the unexploited potential of endophytes as a sustainable source of bioactive compounds. This is important as it provides an alternative to the overharvesting of plants for therapeutic use, placing endophytes as new reservoirs of pharmacological agents. Similarly, in *Polygonatum cyrtonema*, a medicinal plant used in traditional Chinese medicine, microbial structure was found to be directly correlated with the storage of polysaccharides and saponins, which are key compounds to the plant's activity and commercial value (Yang et al.). The endophytic and rhizosphere microbiota differed between the Sichuan and Guangxi provinces, with *Burkholderia, Caballeronia, Paraburkholderia* and *Amycolatopsis* being negatively correlated with levels of bioactive compounds and *Enterobacter* having a positive correlation with the accumulation of polysaccharides. These observations place microbial ecology in the context of not just agronomy, but also the consistency and authenticity of medicinal plant products. In these contexts, the microbiome is no longer a silent background associate and becomes an active driver of cultural, economic, and pharmacological value.

These articles also emphasize the manner in which plant-associated microbiomes support stress tolerance in the main staple crops. Repeated monocropping of peanuts, a key legume, was found to significantly reorganize rhizosphere microbial communities, reducing taxa with beneficial functions in nutrient cycling and disease suppression and increasing potential pathogens. This microbial imbalance, commonly referred to as “continuous cropping obstacles,” contributes to a gradual decrease in peanut yields under prolonged monoculture. Control over microbiome structure was thus found to become a viable option to restore soil equilibrium and maintain productivity in high-input cropping systems. The same was found with rice crops in saline–alkaline soils, where the salt-tolerant cultivar “Jida17” assembled a more diverse and stress-acclimated microbiome compared to the susceptible “Tongxi93,” also showing a positive correlation with improved yield and grain quality (Zhong et al.). Functional pathways involving stress tolerance, nutrient cycling, and hormone modulation were enriched in Jida177-associated communities, illustrating how crop breeding and microbiome engineering are complementary levers for resilience. Casting the net wider to encompass nutrient limitation, phosphate-solubilizing rhizobacteria were found to mobilize phosphorus present in insoluble forms, enhancing rice P status and increasing field yields by as much as 15% (Rasul et al.). The multi-trait functions carried out by *Acinetobacter* MR5 and *Pseudomonas* R7 provided stress relief, suggesting the practical value of well-characterized microbial inocula. All of these studies show that plant productivity from monocultures under salinity and nutrient limitation is associated with the host's ability to recruit, harbor, and work with beneficial microbes.

A study on *Bacillus* sp. SW7 derived from mangroves highlighted an underutilized opportunity of exploring the potential of extremophilic microbes adapted to saline and heat-stressed systems. Isolated from mangrove sediments in the United Arab Emirates (UAE), the bacterium exhibited remarkable tolerance to high salinity (11% NaCl) and temperature (50 °C), and expressed multiple traits typically associated with plant growth-promoting (PGP) characteristics, including: solubilization of phosphate and potassium, production of indole acetic acid and ammonia, and catalase/oxidase activities (Afridi et al.). In a shade-house trial using tomato (*Solanum lycopersicum*), the addition of SW7 to tomato seeds greatly increased seed germination, leaf density and plant biomass. Harvested plants had only slight to no effects on the levels of photosynthetic pigments, indicating that beneficial nutritive substances and stress-tolerance (not photosynthetic efficiency) are responsible for growth-promoting effects. Together the results of this study demonstrate the potential of extremophilic PGP bacteria as bioinoculants for improving agroecological performance in arid and marginal soils, which could allow agriculture to become less reliant on chemical fertilizers and increase its resilience to climate stressors.

In addition to the co-inoculation of PGP bacteria, there are increasing consortia of fungi and bacteria that are being considered for their synergistic roles. Zeng et al. reported that co-inoculation of the arbuscular mycorrhizal fungus (*Funneliformis mosseae*) with a PGP bacterium (*Pseudomanas* sp. SG29) and a PGP rhizobacterium (*Bacillus* sp. SG42) resulted in greater improvements in tobacco seedling growth. Among the treatments tested, A_SG29, a co-inoculation of *F. mosseae* and *Pseudomonas* SG29, produced the greatest changes with regard to biomass, nutrient uptake (N, P, K), leaf area, chlorophyll content and root morphology. Using rhizosphere sequencing, they found evidence of increased beneficial microbial taxa, increased arbuscular mycorrhizal fungi (AMF) root colonization, and upregulation of metabolic pathways related to nutrient cycling and supporting plant growth. These results illustrate the ecological benefits of microbial co-culture: the ability to create targeted combinations of AMF and plant growth-promoting rhizobacteria (PGPR) that shape the microbiome of the rhizosphere enough to enhance early seedling development, while decreasing reliance on agrochemicals.

Soil type itself is a central parameter that affects plant–microbe interactions and their ultimate influence on crop yield responses. For example, in a comprehensive study of ratoon sugarcane (*Saccharum officinarum*) on sandy, loam and clay soils, Wang, Ma et al. determined that soil microbiological functions (which include respiration and catalase activity) were highly correlated with the theoretical sugar yield that could be obtained from the crop. Also, loam soils had the most balanced environment, which supported the greatest rhizosphere function and yield potential compared to sandy or clay soils. Additionally, bacterial abundance in the rhizosphere was negatively correlated with soil biochemical function, while fungal abundance was positively correlated. This indicates the distinct functional roles of fungi and bacteria in sugarcane rhizospheres, with fungi performing more of the biochemical functions associated with yield. The structure of root-associated endophyte communities also differed between soil types, demonstrating its direct effect on plant growth. These findings provide a basis for soil-specific management approaches and highlight the importance of improving the microbial community structure of different soils to increase sugarcane yield in ratoon systems.

While much of the focus has been on crop systems, insights from forest ecosystems can also provide valuable lessons regarding plant-microbe-soil interactions. Guo et al. examined the soil microbiome of the shiro, the distinct mycelial zone of the ectomycorrhizal fungus *Tricholoma bakamatsutake*, in association with *Quercus mongolica*. Shiro soils were found to have higher availability of potassium and nitrogen, but a lower availability of phosphorus and organic matter, when compared to non-mycorrhizal rhizosphere soils. The fungal community was less diverse in shiro soils than in non-mycorrhizal rhizosphere soils, and the community was dominated by *T. bakamatsutake*, which suppressed potential competitors such as *Russula* and *Penicillium*. In contrast, the bacterial community showed more diversity, with enriched communities of mycorrhization-helper taxa (such as *Paenibacillus* and *Bacillus*) and associated plant growth-promoting genera (e.g., *Solirubrobacter* and *Streptomyces*). Functional predictions showed the upregulation of pathways for sugar and fat catabolism the enrichment for genes involved in gibberellin biosynthesis, and carboxylesterase activity.

Microbiomes have also emerged as key players in plant adaptations to environmental contamination. Alongside ecological evidence, experimental work using ectomycorrhizal fungi with *Pinus tabulaeformis* under lead stress showed that fungal partners increase host growth, photosynthesis, and antioxidant defense while immobilizing lead in lead pyromorphite minerals through biomineralization (Cheng et al.). This novel dual mechanism of physiological tolerance on behalf of the host and geochemical stabilization in the rhizosphere is a potential model for the sustainable remediation of contaminated soils. These studies suggest that microbes are highly active participants in contaminated ecological zones, rather than passive residents.

Beyond well-studied bacterial–fungal consortia, other cross-kingdom microbial associations, such as those involving microalgae and bacteria, are emerging as important ecological inputs. An integrated review of microalgae–microbe interactions in saline–alkaline agriculture showed that algal photosynthesis and bacterial respiration constitute metabolic cycles that exchange carbon, oxygen, vitamins, and siderophores, generating extracellular polysaccharides that enhance soil aggregation and water relations (Ren et al.). These consortia were found to promote enhanced plant antioxidant responses and perform better than individual inoculants in soil and foliar treatments. By connecting autotrophic and heterotrophic metabolisms, algal-microbial systems open up new ranges of bioformulations to address salinity stress. Notably, this suggests a future for microbial interventions in agriculture, beyond single-strain inoculants and toward multi-kingdom consortia that reflect the complexity of natural ecosystems.

The microbiomes of shrubs and trees offer a new perspective, emphasizing drought resilience and longevity. A study of the hybrid buffaloberry (*Shepherdia utahensis “Torrey”*) showed that communities of soil, rhizosphere, and nodular organisms, dominated by Proteobacteria, Actinobacteriota, and *Frankia* nitrogen fixers support plant survival in droughty habitats (Devkota et al.). Interestingly, some individual strains had a combination of plant growth-promoting characteristics, including some that displayed all seven functions that were tested. These multipurpose microbes could be advantageous for designing inoculants for low-water-use horticulture and dryland restoration and would be safe from the potential isolation of microbes. An example of this is the study of global citrus root, which identified a conserved core microbiome associated with perennial fruit systems in nine countries and six continents. This microbiome comprised taxa such as *Bradyrhizobium, Pseudomonas, Streptomyces, Cladosporium*, and *Mortierella* (Lombardo et al.). Despite obvious environmental heterogeneity, the consistency of plants and their microbial associations indicates that perennial fruit trees may have a functional template that could be exploited to create universal inoculants. This provides an opportunity for scaling and, by virtue of not needing to adapt to each local environment, suggests that microbiome management involves optimizing conserved cores.

Endophytic microorganisms are part of the plant microbiome and fulfill important functions such as promoting plant growth and protecting against potential pathogens (Ghosh et al., 2020). These mechanisms can be diverse but they have been divided into two categories for study: direct and indirect. Direct mechanisms include functions such as the production of phytohormones and the facilitation of nutrient uptake from the soil. On the other hand, indirect mechanisms include the production of antimicrobial compounds that inhibit pathogen growth and the stimulation of the plant's defense systems (Glick and Gamalero, 2021). By providing these microbial services to their plant host, plants were found to exhibit better growth and to be able to improve their overall fitness even under biotic or abiotic stress conditions (e.g., drought or salinity) (Kumar and Nautiyal, 2022).

Two microbial groups reported as endophytes are fungi of the genus *Trichoderma* and bacteria of the genus *Bacillus*. Santoyo et al. analyzed the multitrait characteristics of these two groups. While their work did not exclusively focus on endophytes, they did emphasize the importance of these groups in performing synergistic tasks that help plants tolerate salt stress. Salt stress in agriculture is a global problem affecting a large portion of arable land, approximately 40%, and is more severe in underdeveloped regions.

Another issue affecting arable land is the presence of heavy metals and metalloids such as arsenic (Zhou et al., 2018). However, it has been shown that endophytic fungi can increase tolerance to these contaminants and even aid in the phytoremediation of contaminated soils. Such is the case of the endophytic fungus *Serendipita indica*, which in a synergistic interaction with the actinobacterium *Zhihengliuella* sp. ISTPL4 improved rice tolerance to arsenic presence and toxicity. Both microorganisms improved plant growth parameters such as shoot length, root length, shoot dry weight, and root dry weight, in addition to biochemical parameters such as chlorophyll content, protein content, and antioxidant enzymatic activities. These improvements were observed in comparison to uninoculated control plants. Additionally, plants managed to withstand arsenic stress due to the increased production of phytohormones in the presence of the microbial mixture (Sharma et al.).

The study of beneficial microorganisms can be approached using two strategies: culture-based and non-culture-based. The former provides an opportunity to study or manipulate the microorganisms in interaction with various crops, and also offers the possibility of generating bioinoculants applicable to different production systems. For example, Safaie et al. isolated over 1,000 strains of endophytic fungi from plant tissues such as roots and shoots (from *Ferula ovina, F. galbaniflua*, and *F. persica*), which were assigned to different species within the orders Eurotiales, Pleosporales, Botryosphaeriales, Cladosporiales, Helotiales, Hypocreales, Sordariales, Glomerellales, and Polyporales. Interestingly, root tissues harbored greater diversity than aerial tissues, given their closer contact with the rhizosphere.

Using techniques such as high-throughput 16S rRNA gene sequencing, the diversity of endophytic bacterial populations was determined in three Sichuan bamboo species: *Phyllostachys edulis, Bambusa rigida*, and *Pleioblastus amarus*. Out of a total of 1,159 operational taxonomic units (OTUs) possibly belonging to 811 species, the most abundant phyla were Proteobacteria, Actinobacteria, and Myxococcota. According to a functional analysis using PICRUSt, endophytic bacteria in bamboo leaves are mainly associated with six biological pathways: human diseases, metabolism, cellular processes, environmental and genetic information processing, and organ systems. These results indicate that their metabolic functions jointly influence the genetics, environment, and community structure of bamboo (Yan et al.).

The microbial diversity associated with a plant can be influenced by various factors, including biotic stress—particularly from pathogen attacks—which can lead to the recruitment of beneficial microbes (Liu et al., 2021). Wang, Xu et al. evaluated the endophytic microbiome of walnut (*Juglans regia*) in interaction with two pathogens: *Colletotrichum gloeosporioides* and *Fusarium proliferatum*. The results showed that despite changes in relative abundances, the dominant bacterial communities remained similar during infection by both pathogens. Interestingly, endophytic fungi were more sensitive to the presence of pathogens. However, both pathogens, *C. gloeosporioides* and *F. proliferatum*, promoted the enrichment of beneficial bacteria such as *Bacillus* and *Pseudomonas*, which are widely reported as antagonists of pathogenic fungi and promoters of plant growth. Their study also evaluated the performance of the endogenous antagonistic bacteria *Pseudomonas psychrotolerans* and *Bacillus subtilis*, which exhibited inhibitory effects on both pathogenic fungi and participated in the interaction.

The role of endophytes in challenging environments has been increasingly documented. Wang, Shi et al. provided a comprehensive analysis of dark septate endophytes (DSEs) in plant roots in heavy metal-contaminated ecosystems, identifying 22 distinct DSE species and revealing that colonization patterns are profoundly shaped by environmental variables such as soil nutrient status, organic matter content, and overall fungal community diversity. Their research group demonstrated that nutrient-rich soils foster higher DSE abundance, whereas heavy metal stress selects for resilient fungal communities with enhanced tolerance. This research not only elucidates the ecological determinants of DSE distribution but also establishes a theoretical foundation for leveraging these endophytes in the bioremediation of polluted habitats.

In a complementary study, Dong et al. examined the role of native seed endophytes in tobacco's resistance to *Ralstonia solanacearum*. High-throughput sequencing uncovered a marked increase in *Paenibacillus* in the resistant varieties, and the isolated strain *Paenibacillus odorifer* 6036-R2A-26 was found to effectively suppress bacterial wilt while simultaneously promoting plant growth. These results emphasize the dual functional potential of seed-associated microbiomes in enhancing both disease resistance and crop productivity, offering new avenues for microbiome-informed breeding and biocontrol strategies.

Understanding the drivers of rhizosphere community assembly is another key research focus. Ma et al. investigated the assembly of rhizosphere microbial communities in 108 plant samples by integrating the analysis of root traits, soil chemistry, enzyme activity, and metabolite profiles. The authors revealed that small-molecule metabolites—such as glycerol, sorbitol, phytol, and α-ketoglutaric acid—serve as primary drivers of microbial community composition, exerting a greater influence than either soil physicochemical properties or root morphological traits. *Rhizobium* emerged as a keystone genus shaping community structure, and experimental supplementation with these metabolites successfully steered microbial assemblages toward configurations enriched with beneficial microbes. These findings highlight the transformative potential of targeted metabolite interventions in engineering plant-associated microbiomes for improved ecosystem function and crop performance.

The integration of natural compounds with beneficial microbes provides innovative solutions. Jiang et al. explored the synergistic effects of osthole and *Bacillus amyloliquefaciens* on *Panax quinquefolius* in a forest ecosystem. Their treatments enhanced photosynthetic efficiency, upregulated antioxidant enzyme activity, and modulated the root microbial community by increasing bacterial diversity while reducing fungal diversity. Crucially, the recruitment of growth-promoting microbes resulted in increased biomass and a decreased incidence of anthracnose, demonstrating the practical potential of combining natural bioactive compounds with beneficial microbes to elevate plant resilience and productivity in the field.

Traditional agroecosystems continue to reveal valuable microbial resources. Rivera-Hernández et al. characterized the culturable rhizobacterial communities of tunicate maize grown in traditional Mexican agroecosystems and revealed stage-specific functional roles, with tasseling-stage bacteria enhancing growth and pathogen suppression, and maturity-stage bacteria contributing to organic matter mineralization and nutrient cycling. Importantly, several genera, including *Rhizobium, Sphingobium*, and *Arthrobacter*, were identified as previously undescribed plant growth-promoting rhizobacteria (PGPR) in maize landraces. This work highlights the critical importance of conserving native crops and their associated microbial genetic resources as reservoirs for sustainable agriculture and novel biotechnological applications.

## Conclusion

In conclusion, plants and their microbiomes function as integrated holobionts whose interactions are essential for evolution, ecosystem balance, and agricultural resilience. Understanding and harnessing these partnerships offers key solutions to challenges in food security, environmental stress, and sustainable resource use.
